# Supplementary material for: Blautia coccoides JCM1395T Achieved Intratumoral Growth with Minimal Inflammation: Evidence for Live Bacterial Therapeutic Potential by an Optimized Sample Preparation and Colony PCR Method
Source: Pharmaceutics. 2023 Mar 19;15(3):989. doi: 10.3390/pharmaceutics15030989 (PMC10058202; doi:10.3390/pharmaceutics15030989)
Supplement: Supplementary file 1 [file pharmaceutics-15-00989-s001.zip › pharmaceutics-2207962-supplementary.pdf]

Supplementary Fig. S1

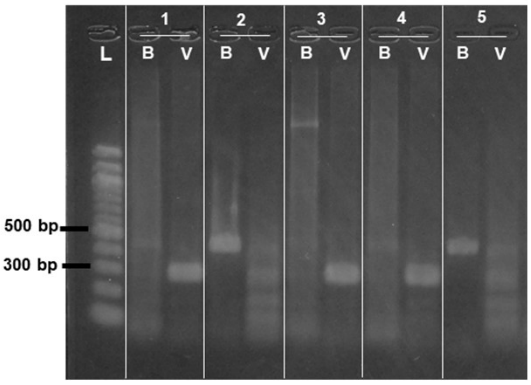

Agarose gel electrophoresis images with 16S rRNA gene PCR amplicons of five colonies picked from an agar plate seeded with a mixture of *B. coccoides* and *B. vulgatus*. PCR amplicons by primers corresponding to *B. coccoides* and *B. vulgatus* were loaded in lane B and lane V, respectively. The number in the figure indicates each colony.

Supplementary Fig. S2

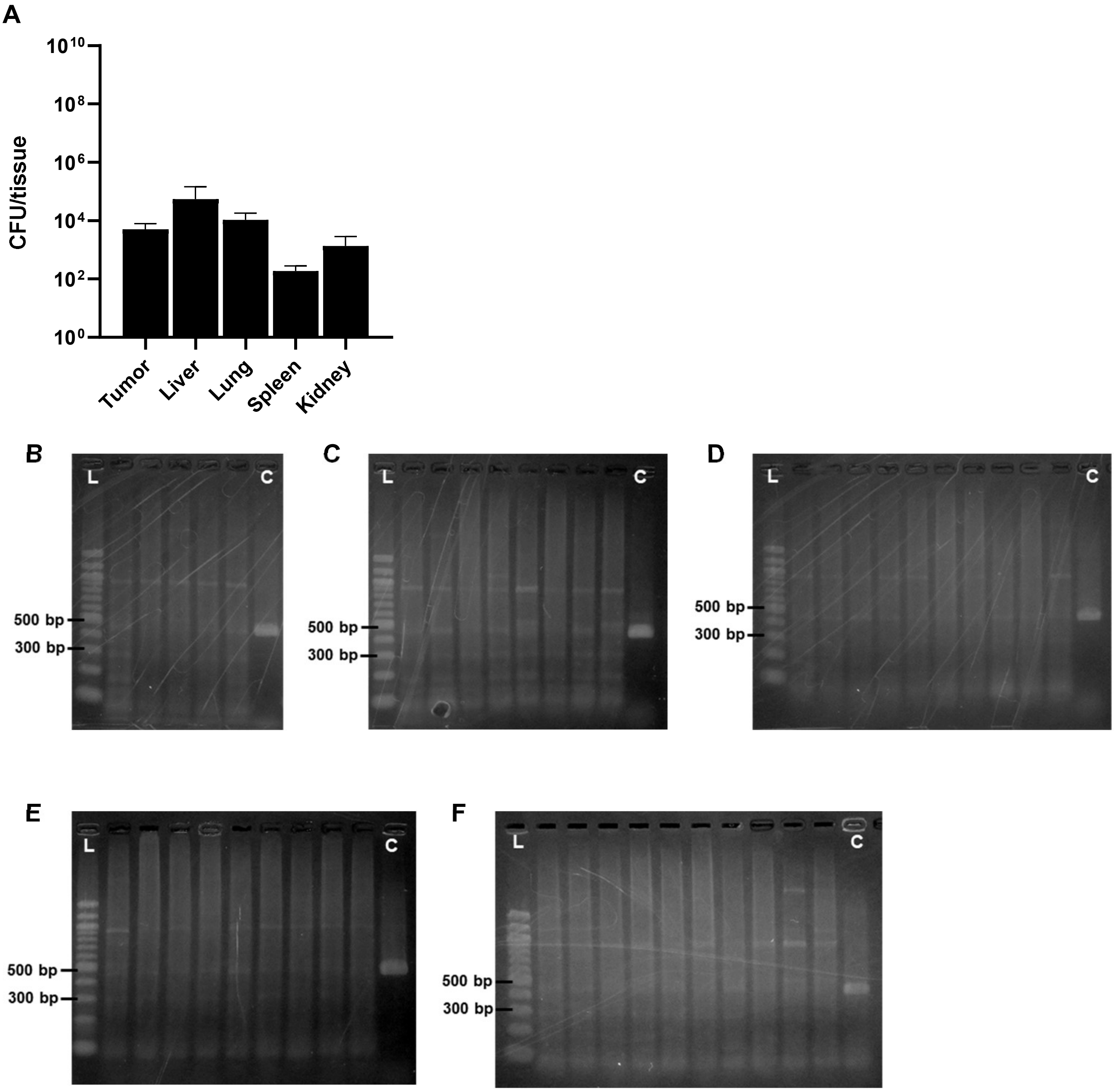

The number of anaerobic bacteria originally present in the tissues of tumor-bearing mice. (A) CFUs of bacteria on the agar plates seeded with homogenates of tumors and major organs and cultured anaerobically. Each value represents the mean ± standard deviation (SD) (n = 3). (B–F) SYBR® GREEN stained agarose gel electrophoresis images applied with 16S rRNA gene PCR amplicon by primers for *B. coccoides*. Colonies were picked from agar plates seeded with homogenates of (B) tumors (× 10 dilutions), (C) kidney (× 10 dilutions), (D) lung (× 100 dilutions), (E) liver (× 100 dilutions), and (F) spleen (× 10 dilutions). PCR amplicons of cultured *B. coccoides* were loaded in lane C as a positive control.

Supplementary Fig. S3

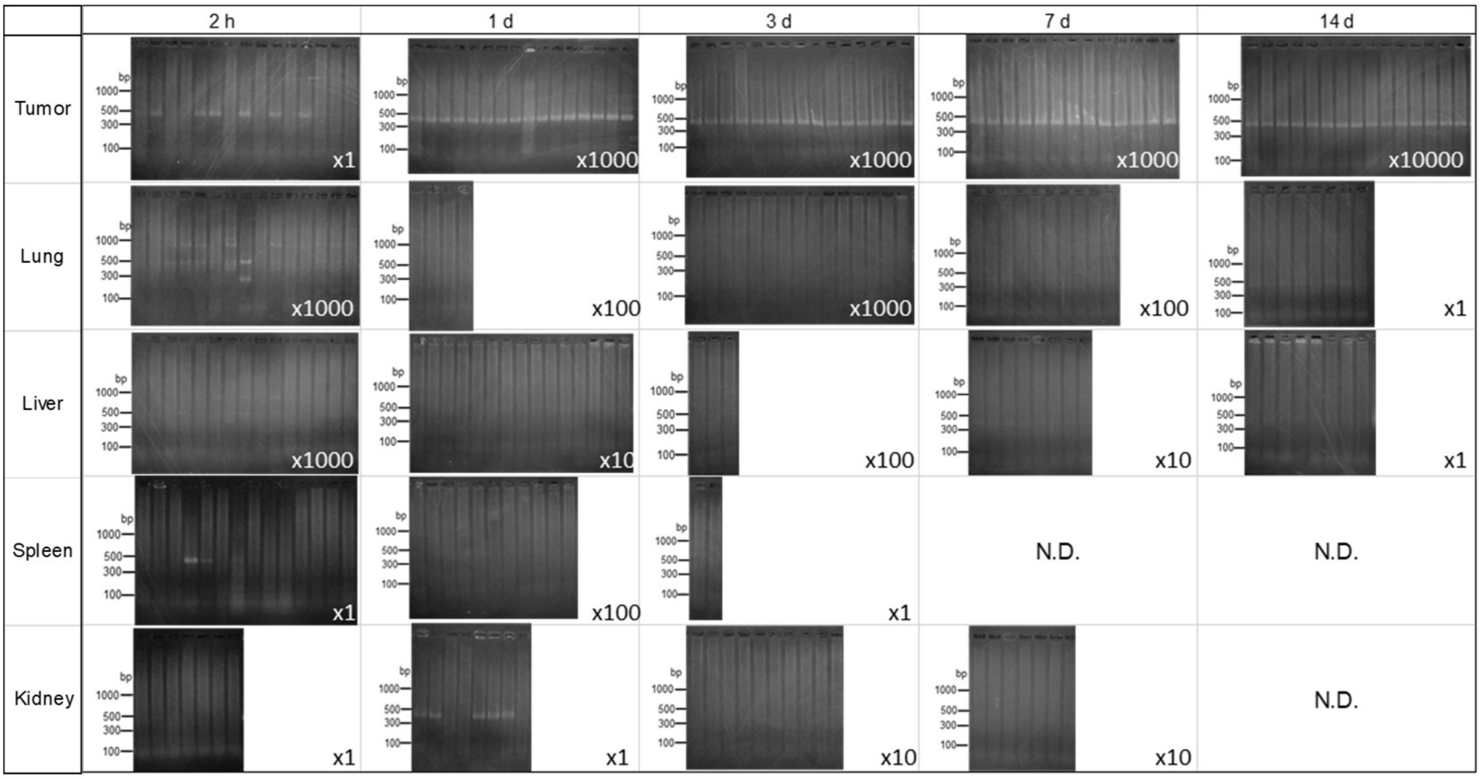

SYBR® GREEN stained agarose gel electrophoresis images of 16S rRNA gene PCR amplicons of colonies picked from agar plates seeded with diluted tissue homogenates. Tumors and tissues were extracted from colon-26 tumor-bearing mice receiving *B. coccoides* (1.5×10^7 CFU). The numbers at the bottom right indicate the dilution factor of the homogenate of each organ.
